# Supplementary material for: Abundance-biased codon diversification prevents recombination in AAV production and ensures robust in vivo expression of functional FRET sensors
Source: Commun Biol. 2025 Aug 19;8:1244. doi: 10.1038/s42003-025-08677-6 (PMC12365220; doi:10.1038/s42003-025-08677-6)
Supplement: Supplementary file 1 — Supplementary Information [file 42003_2025_8677_MOESM1_ESM.pdf]

Abundance-Biased Codon Diversification prevents recombination in AAV production and ensures robust in vivo expression of functional FRET sensors

Jan Dernic[1,3,#], Afroditi Eleftheriou[1,3,#], Lazaros Vasilikos[2], Melanie Rauch[2], Pascal Imseng[1,3], Henri Zanker[1,3], Zoe J. Looser[1,3], Rachel M. Meister[1,3], Felipe Velasquez Moros[1,3], Tomer Kagan[4], Tal Laviv[4,5], Jean-Charles Paterna[2], Michael Arand[1], Aiman S. Saab[1,3], Bruno Weber[1,3], Luca Ravotto[1,3,*]

[#]These authors contributed equally

[1] Institute of Pharmacology and Toxicology, University of Zurich, Zurich, Switzerland;

[2] Viral Vector Facility, University of Zurich and Swiss Federal Institute (ETH) Zurich, Zurich, Switzerland;

[3] Neuroscience Center Zurich, University and ETH Zurich, Zurich, Switzerland;

[4] Department of Physiology and Pharmacology, Gray Faculty of Medical and Health Sciences, Tel Aviv University, Tel Aviv;

[5] Sagol School of Neuroscience, Tel Aviv University, Tel Aviv, Israel;

Table of Contents

Figure SI1: DNA sequencing analysis for FLIIP/cdFLIIP 3

Figure SI2: DNA sequencing analysis for ATeam/cdATeam 5

Figure SI3: DNA sequencing analysis of starting plasmids for FLIIP and ATeam 7

Figure SI4: Next-generation sequencing analysis of recombination for FLIIP and ATeam....8

Figure SI5: Southern blots for cdFLIIP and cdATeam, confirmation of functionality for cdATeamDA....... 9

Figure SI6: Sparse neuronal targeting via i.v. injection of AAV-PhP.eb cdFLIIP....... 10

Figure SI7: DNA sequencing analysis for hERT2-Cre-hERT2/hERT2-Cre-cdhERT2....... 11

Figure SI8: Codon Diversification of the G-PTEN phosphatase sensor....... 12

Figure SI9: Uncropped gels 13

Tables SI1-SI3: Objective function values for codon diversification runs under different conditions....... 13

Supplementary Note 1: Codon diversified sequences for proteins in this study....... 14

Figure SI1: DNA sequencing analysis for FLIIP/cdFLIIP

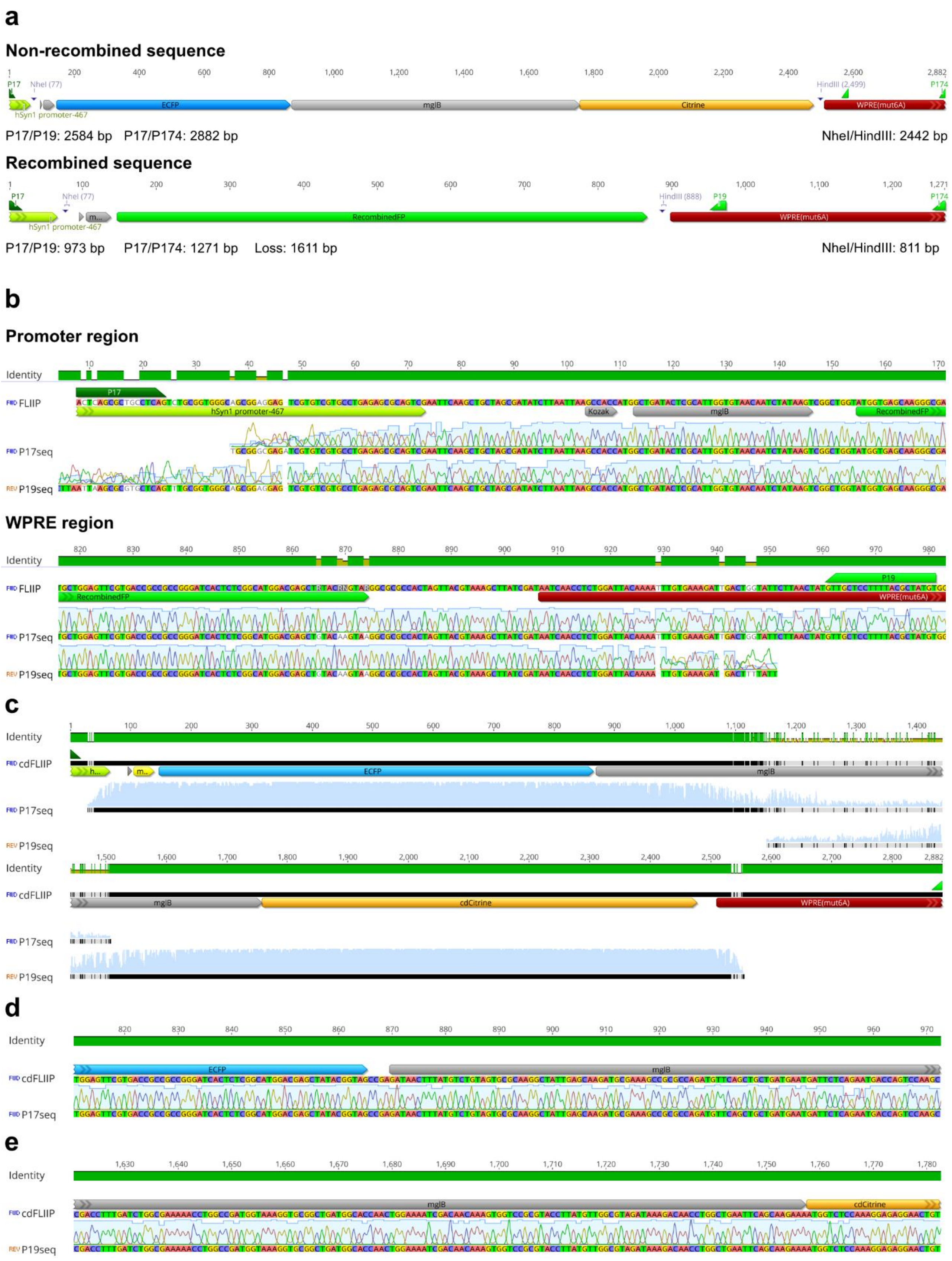

Figure SI1. DNA sequencing analysis for FLIIP/cdFLIIP: (a) Sequences of the amplified DNA stretches for analysis, for the non-recombining and recombining FLIIP sensor. Sequence lengths for stretches between relevant primers (sequencing and gel electrophoresis) or restriction sites (Southern blot) are reported together with the number of nucleotides lost during recombination. (b) Sequencing results for FLIIP, demonstrating the presence of a single recombined FP instead of the full sensor. (c) Overview of the sequencing results for cdFLIIP, highlighting the presence of the glucose binding unit, and the fact that recombination was prevented. (d, e) Zoomed view of the sequencing results in (c), focusing on the regions encompassing the binding unit and the two fluorescent proteins.

Figure SI2: DNA sequencing analysis for ATeam/cdATeam

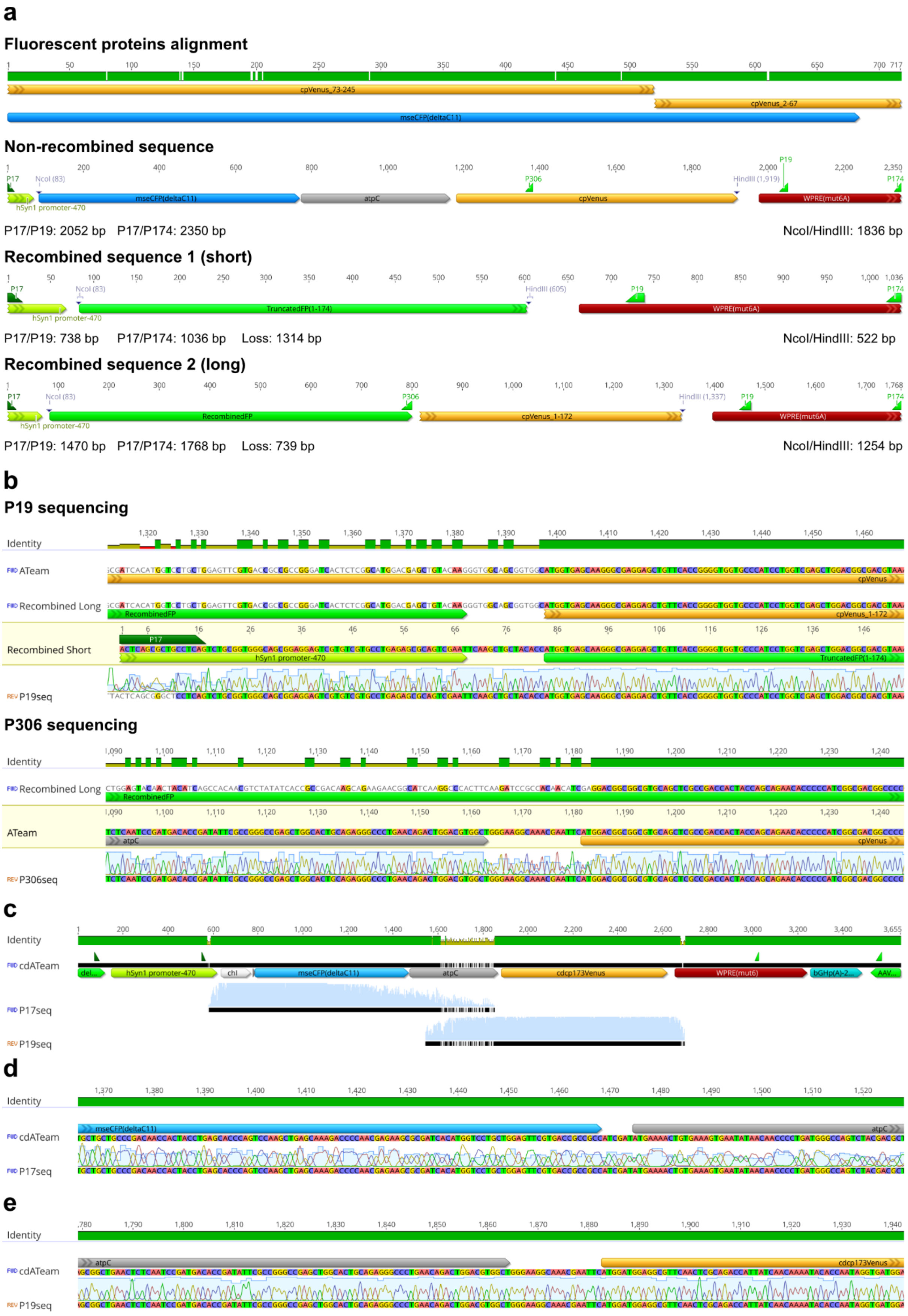

Figure SI2. DNA sequencing analysis for ATeam/cdATeam: (a) Sequences of the amplified DNA stretches for analysis, for the non-recombining Ateam1.03 sensor and the two possible recombination outcomes. Sequence lengths for stretches between relevant primers (sequencing and gel electrophoresis) or restriction sites (Southern blot) are reported together with the number of nucleotides lost during recombination. (b) Sequencing results for ATeam, demonstrating the presence of a large amount of the "short" recombined product, together with a minor fraction of fully formed sensor (P19). By sequencing using a primer that does not bind to the "short" recombined sequence (P306), we show that the major component is the fully formed sensor, with a minor amount of the "long" recombined sequence. Southern blot was used to confirm this quantitative trend (Figure 3b). (c) Overview of the sequencing results for cdATeam, highlighting the presence of the ATP binding unit, and the fact that recombination was prevented. (d, e) Zoomed view of the sequencing results in (c), focusing on the regions encompassing the binding unit and the two fluorescent proteins.

Figure SI3: DNA sequencing analysis of starting plasmids for FLIIP and ATeam

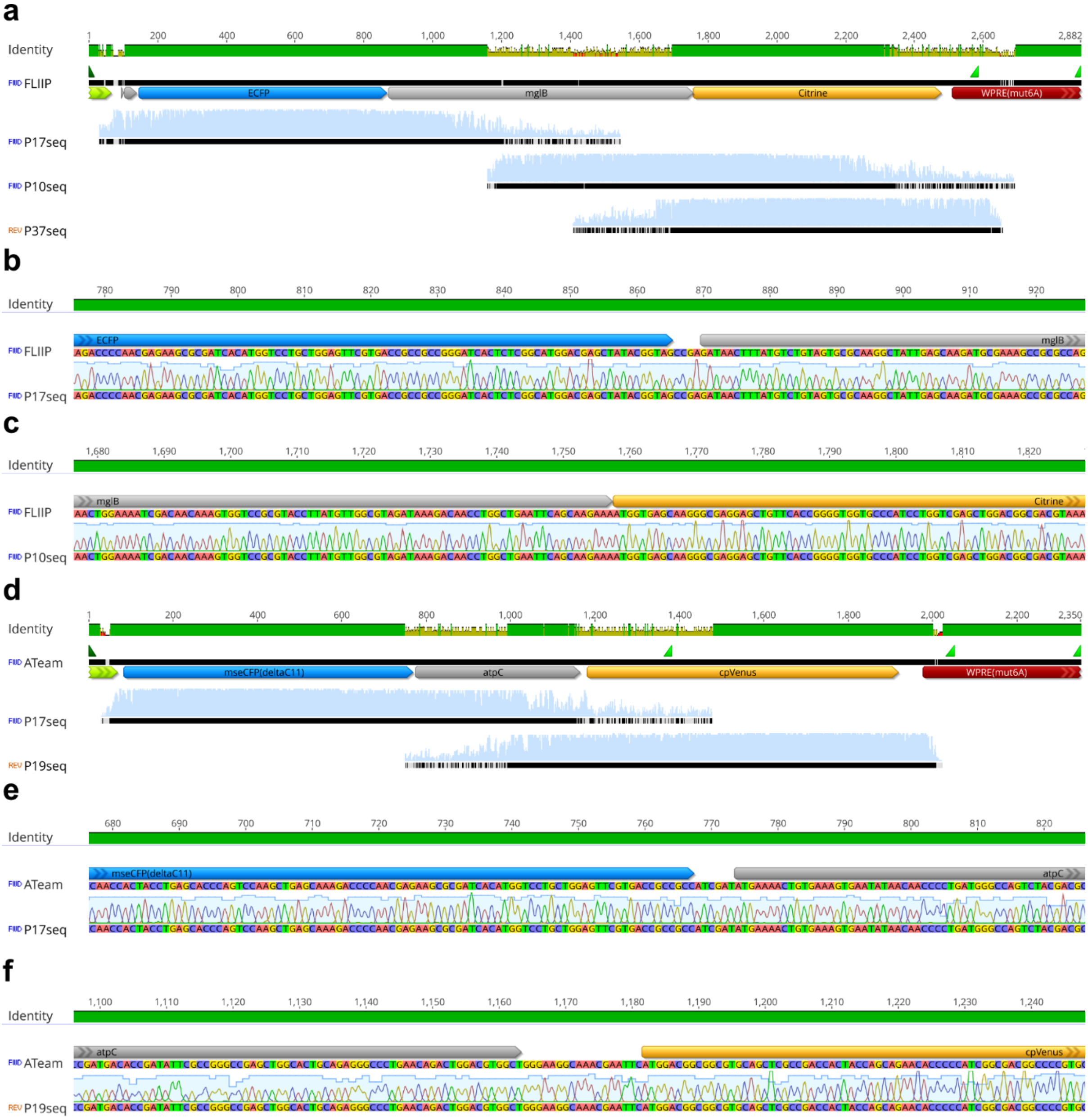

Figure SI3. DNA sequencing analysis of starting plasmids for FLIIP and ATeam: (a,d) Overview of the sequencing results for the plasmids used as starting material for AAV preparations for FLIIP (a) and ATeam (d), showing no recombination. (b, c, e, f) Zoomed view of the sequencing results for FLIIP (b,c) and ATeam (e,f), focusing on the regions encompassing the binding unit and the two fluorescent proteins.

Figure SI4: Next-generation sequencing analysis of recombination for FLIIP and ATeam

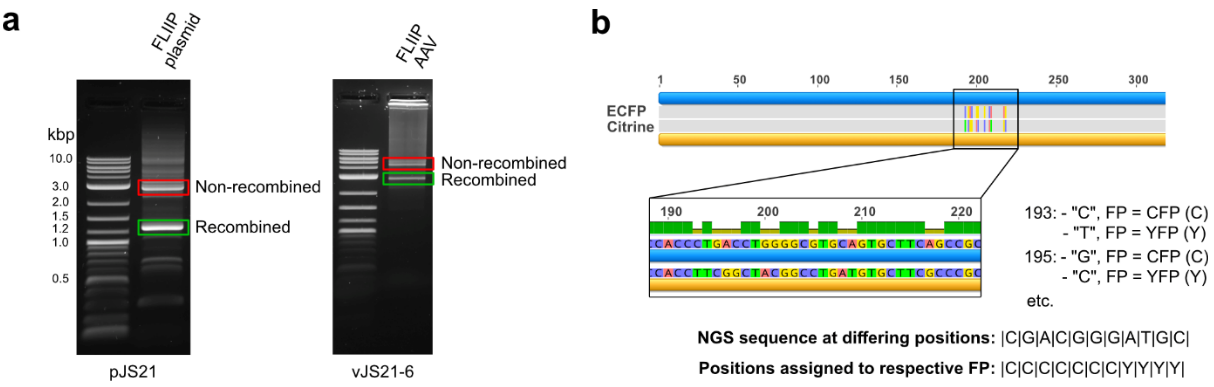

c

FLIIP_pJS21 (Recombined)	Abundance [%]
CCCCCCCCCCCYYYYYYYYYYY	34.95
YYYYYYYYYYYYYYYYYYYYYY	20.28
CCCCCCCCCCCCCCCCCCYYYY	13.76
CCCCCCCCCCCCCCCCCYYYYY	12.47
CCCCCCCCCCCCYYYYYYYYYY	4.3
CCCCCCCCCCCCCCYYYYYYYY	3.92
CCCCCCCCCYYYYYYYYYYYYY	1.08

FLIIP_vJS21-6 (Non recombined)	Abundance [%]
CCCCCCCCCCCCCCCCCCCCCC	96.83
Other (seq. errors)	< 0.44

FLIIP_vJS21-6 (Recombined)	Abundance [%]
CCCCCCCCCCCYYYYYYYYYYY	35.86
YYYYYYYYYYYYYYYYYYYYYY	23.57
CCCCCCCCCCCCCCCCCYYYYY	18.98
CCCCCCCCCCCCCCCCCCYYYY	12.94
CCCCCCCCCCCCCCYYYYYYYY	1.32
CCCCCCCCCCCCYYYYYYYYYY	1.09

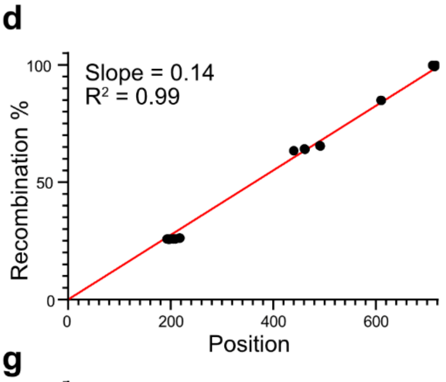

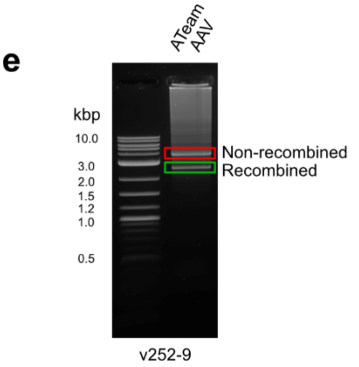

f

ATeam_v252-9 (Recombined)	Abundance [%]
CCCCCCCCCYY	39.53
CCCCCCCCYYY	17.97
YYYYYYYYYYY	12.99
CYYYYYYYYYY	9.17
CCCCCCCCCCY	7.29
CCCYYYYYYYY	5.68
CCCCCCCCCCC	3.83

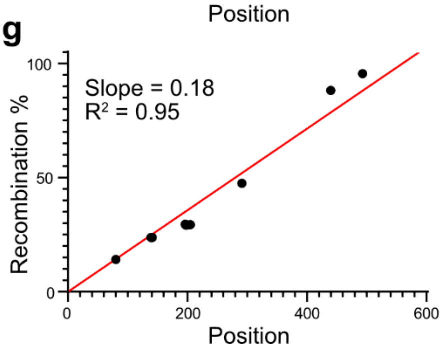

Figure SI4. Next-generation sequencing analysis of recombination for FLIIP and ATeam: (a) Gel electrophoresis of the PCR-amplified starting plasmid (left) and thermally denaturated (non-amplified) viral vectors (right) for FLIIP, demonstrating that recombination occurs both during PCR and AAV preparation. (b) Scheme of our assignment algorithm. We created new sequences containing only the nucleotides at positions in which the sequence differs between CFPs and YFPs, then we assigned each nucleotide to the respective fluorescent protein. In this way, for each NGS read, we could reconstruct where recombination occurred. (c) Abundance of different sequences, for NGS analysis on the recombined band of PCR-amplified plasmid or thermally denaturated AAVs for FLIIP. The non-recombined band of the latter was also analyzed to determine the threshold above which the results can be considered reliable. (d) Percentage of recombination as function of the position in the sequence for FLIIP, showing that recombination increases linearly with distance from the N-term. (e,f,g) Same as (a,c,d), for ATeam.

Figure SI5: Southern blots for cdFLIIP and cdATeam, confirmation of functionality for cdATeamDA

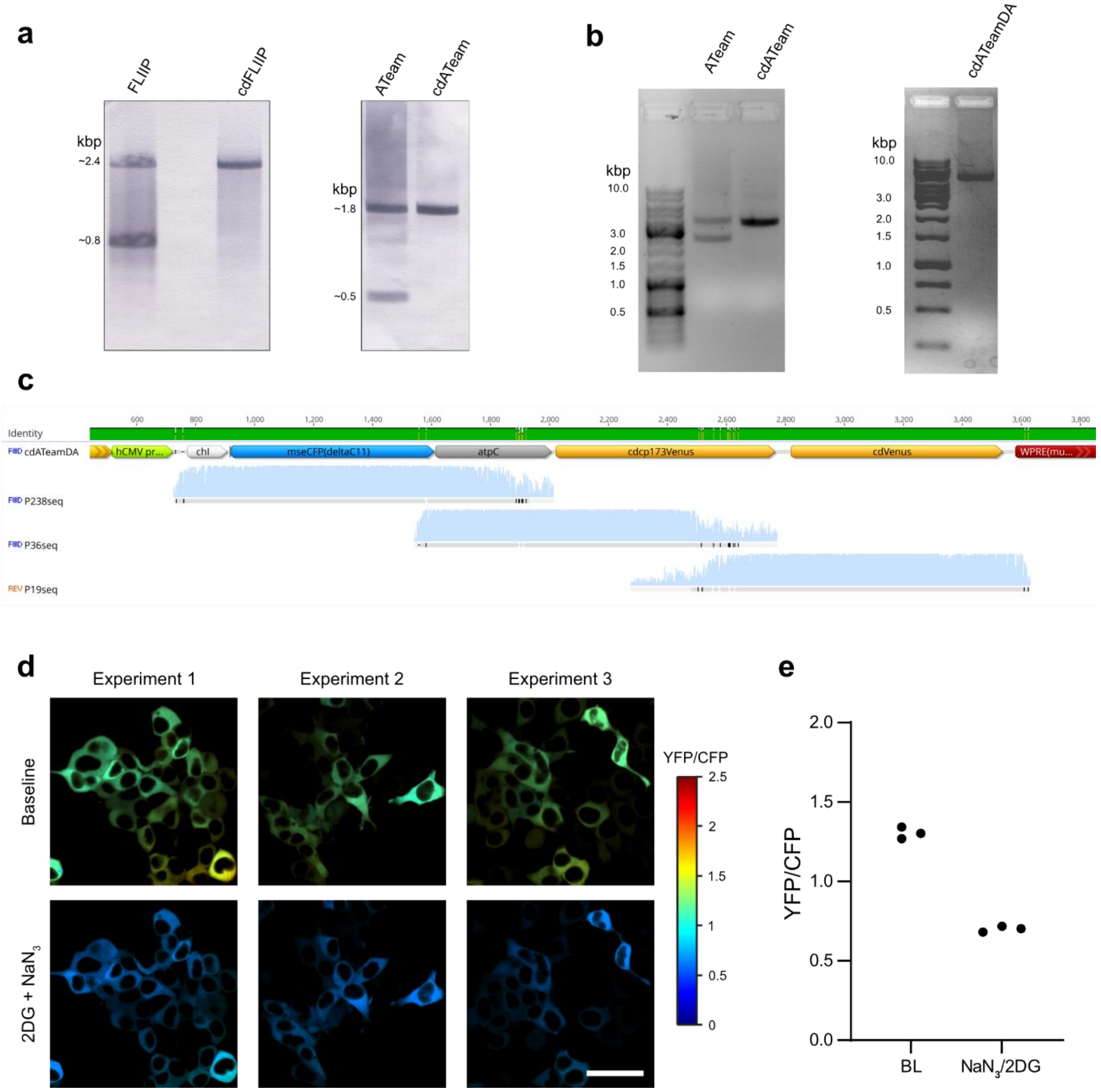

Figure SI5. Southern blots for cdFLIIP and cdATeam, confirmation of functionality for cdATeamDA: (a) Southern blots of the viral vector DNAs, confirming the presence of a large fraction of recombined DNA in the original sensors (~75% for FLIIP, ~66% for ATeam), which is completely absent in the codon-diversified analogues. (b) Gel electrophoresis of thermally denaturated AAV preparations for ATeam, cdATeam and cdATeamDA, showing the lack of recombination in the codon diversified constructs. (c) Sequencing results for PCR-amplified AAV preparations of cdATeamDA, confirming that the correct construct is present in the viral vectors. (d) Two-photon excited ratiometric images for three experimental repeats of an ATP-depleting protocol, for cdATeamDA expressed in HEK cells via viral transduction. Upon 30-min incubation in presence of the metabolic blockers 2-deoxyglucose (2DG, 10 mM) and sodium azide (NaN_3, 10 mM), the sensor shows a significant decrease of the YFP/CFP ratio, confirming its functionality. Scale bar = 35 μm. (e) Ratio values (whole frame average) for the experiments in (d).

Figure SI6: Sparse neuronal targeting via i.v. injection of AAV-PhP.eb cdFLIIP

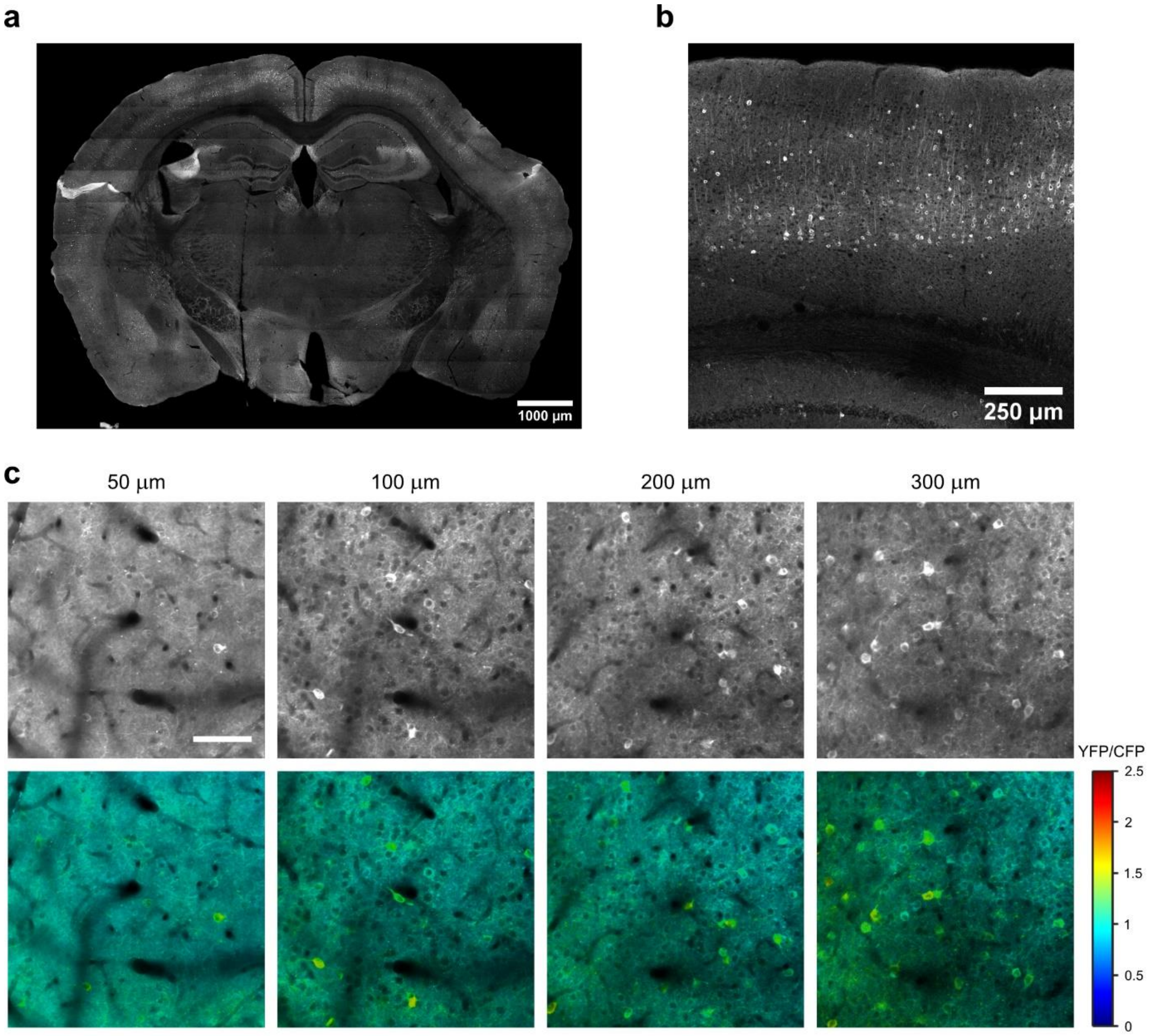

Figure SI6. Sparse neuronal targeting via i.v. injection of AAV-PhP.eb cdFLIIP: (a) Confocal microscopy image of coronal brain sections of a mouse injected with AAV-PHP.eB vectors containing neuronal targeted cdFLIIP, stained with anti-GFP antibodies. Expression of the sensor is visible across the entire cortex and on several other brain areas. (b) Zoomed view of a cortical area of (a), highlighting the sparse neuronal expression. (c) Two-photon excited intensity and ratiometric images acquired at different cortical depths in a mouse injected with AAV-PHP.eB-hSyn-cdFLIIP. Scale bar = 50 µm.

Figure SI7: DNA sequencing analysis for hERT2-Cre-hERT2/hERT2-Cre-cdhERT2

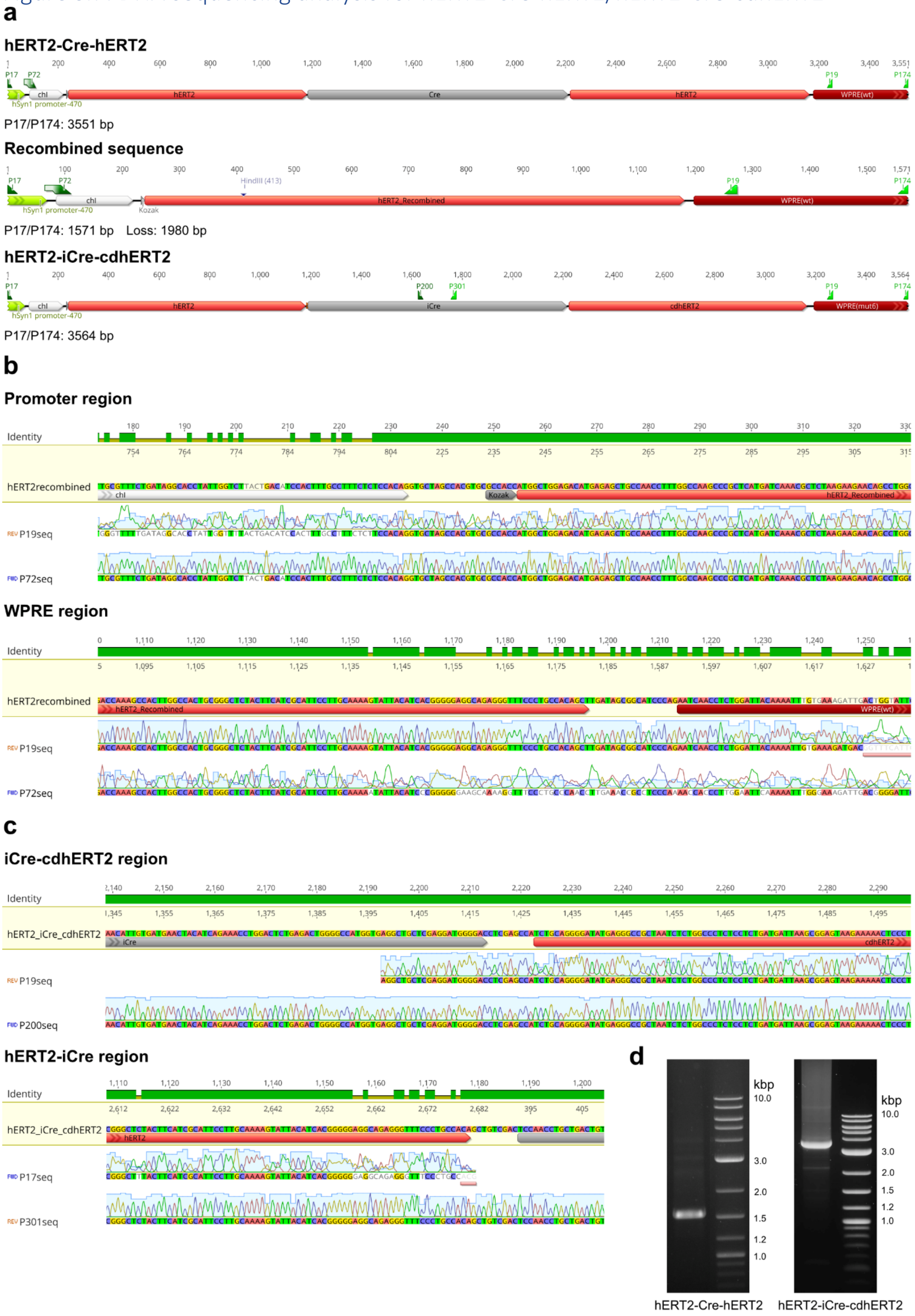

Figure SI7. DNA sequencing analysis for hERT2-Cre-hERT2/hERT2-Cre-cdhERT2: (a) Sequences of the amplified DNA stretches for analysis, for the non-recombining and recombining hERT2-Cre-hERT2. Sequence lengths for stretches between relevant primers (sequencing) are reported together with the number of nucleotides lost during recombination. (b) Sequencing results for hERT2-Cre-hERT2, demonstrating the presence of a single recombined hERT2 instead of the full construct. (c) Sequencing results for hERT2-iCre-cdhERT2, demonstrating the presence of the Cre unit, a clear sign that recombination was prevented (d) Gel electrophoresis of the PCR-amplified viral vector DNAs, showing nearly exclusively a recombination band for the hERT2-Cre-hERT2 construct and the absence of recombination for the codon diversified hERT2-iCre-cdhERT2.

Figure SI8: Codon Diversification of the G-PTEN phosphatase sensor

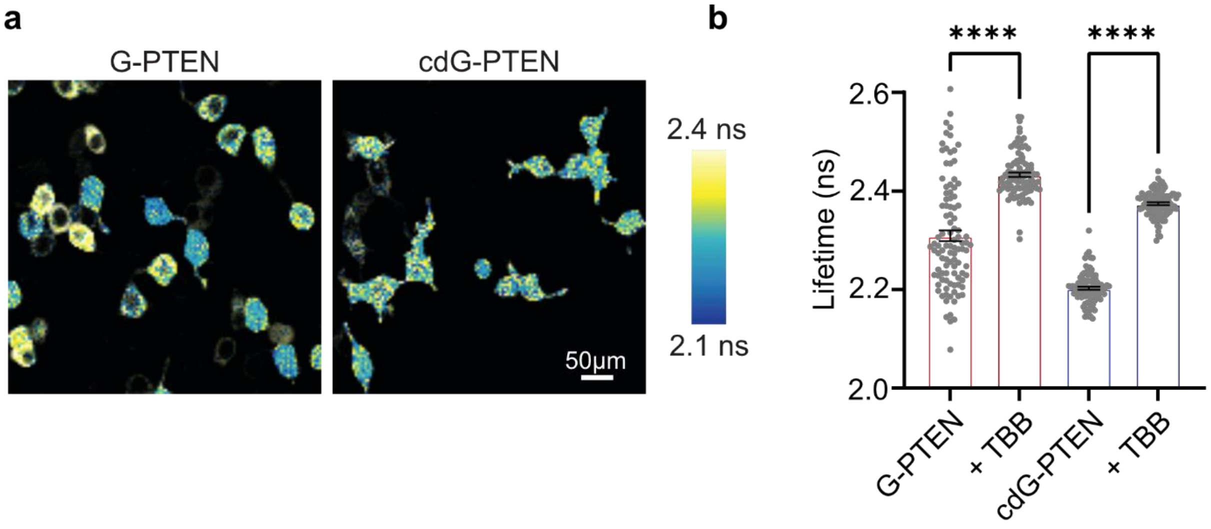

Figure SI8. Codon Diversification of the G-PTEN phosphatase sensor: (a) Representative images of HEK293 cells expressing AAV of GPTEN or cd-G-PTEN. Scale bar, 50 μm. (b) Quantification of mean fluorescence lifetime of G-PTEN (****, 2.31 ± 0.011 ns, *n* = 100, and 2.433 ± 0.004 ns, *n* = 99) or cdG-PTEN (****, 2.20 ± 0.003 ns, *n* = 100, and 2.37 ± 0.003 ns, *n* = 100), before and after 2 hr of TBB (50 μM) application. 'n' denotes number of cells, error bars represent s.e.m. Statistical differences were measured using one-way ANOVA followed by post-hoc Tukey's multiple comparison test. **** $p < 0.0001$. Coefficient of variation: AAV GPTEN = 4.751%; AAV GPTEN + TBB = 1.813%; AAV CD GPTEN = 1.422%; AAV CD GPTEN + TBB = 1.110%. Both the images and the statistical analysis show a much reduced variability of the sensor in absence of TBB, while in the TBB experiments the fact that the "little-quenched" EGFP is similar to the recombination product (a mixture of GFPs) strongly reduces the variability.

Figure SI9: Uncropped gels

a

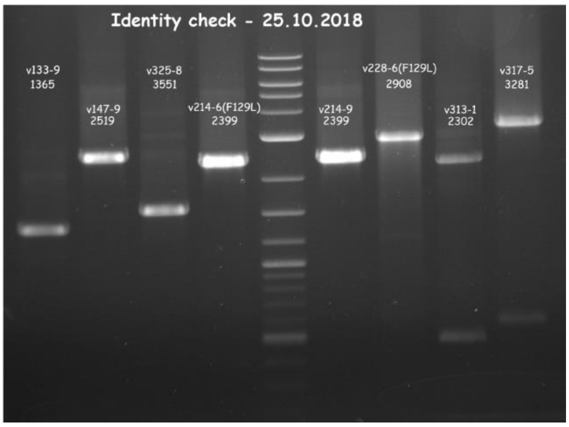

Figure SI9. Uncropped gels: (a) Figure SI7d left. The construct is identified with the label v325-8.

Tables SI1-SI3: Objective function values for codon diversification runs under different conditions

FP pair	*f* value	FP pair	*f* value
EGFP_mTFP1	1.03×10^{10}	mTFP1_mStayGold	8.55×10^{7}
EGFP_mRuby	4.16×10^{11}	mRuby_mCherry	2.88×10^{10}
EGFP_mCherry	1.03×10^{10}	mRuby_mNeonGreen	4.21×10^{6}
EGFP_mNeonGreen	8.72×10^{6}	mRuby_mStayGold	5.86×10^{6}
EGFP_mStayGold	1.35×10^{7}	mCherry_mNeonGreen	1.03×10^{8}
mTFP1_mRuby	6.44×10^{10}	mCherry_mStayGold	1.09×10^{8}
mTFP1_mCherry	5.91×10^{13}	mNeonGreen_mStayGold	6.85×10^{5}
mTFP1_mNeonGreen	8.34×10^{7}		

Table SI1: Objective function values for similarity runs for FP pairs coming from different organisms, using the sequences commonly used for mammalian expression.

	**cdCitrine**	**cdcp173Venus**	**cdcp173tdVenus**
**Codon Bias 0%**	8.49×10^{6}	8.73×10^{6}	8.76×10^{6}
**Codon Bias 5%**	8.49×10^{6}	8.73×10^{6}	8.76×10^{6}
**Codon Bias 10%**	8.49×10^{6}	8.73×10^{6}	8.77×10^{6}
**Codon Bias 15%**	8.49×10^{6}	8.73×10^{6}	8.78×10^{6}
**Codon Bias 20%**	8.52×10^{6}	8.77×10^{6}	2.34×10^{8}
	**ECFP cdCitrine**	**mseCFPΔ11 cdcp173Venus**	**mseCFPΔ11 cdcp173tdVenus**
**Codon Bias 0%**	8.53×10^{6}	8.55×10^{6}	8.58×10^{6}
**Codon Bias 5%**	8.53×10^{6}	8.55×10^{6}	8.58×10^{6}
**Codon Bias 10%**	8.53×10^{6}	8.55×10^{6}	8.59×10^{6}
**Codon Bias 15%**	8.54×10^{6}	8.55×10^{6}	8.61×10^{6}
**Codon Bias 20%**	8.57×10^{6}	8.59×10^{6}	2.33×10^{8}

Table SI2: Objective function values for diversification and similarity runs using the ABCD approach with exclusion of target sequences for ITRs, restriction sites and bacterial IRBs.

	cdCitrine	cdcp173Venus	cdcp173tdVenus
**Codon Bias 0%**	$8.49x10^{6}$	$8.73x10^{6}$	$8.74x10^{6}$
**Codon Bias 5%**	$8.49x10^{6}$	$8.73x10^{6}$	$8.74x10^{6}$
**Codon Bias 10%**	$8.49x10^{6}$	$8.73x10^{6}$	$8.75x10^{6}$
**Codon Bias 15%**	$8.49x10^{6}$	$8.73x10^{6}$	$8.76x10^{6}$
**Codon Bias 20%**	$8.52x10^{6}$	$8.77x10^{6}$	$1.68x10^{7}$
	**ECFP cdCitrine**	**mseCFPΔ11 cdcp173Venus**	**mseCFPΔ11 cdcp173tdVenus**
**Codon Bias 0%**	$8.53x10^{6}$	$8.55x10^{6}$	$8.55x10^{6}$
**Codon Bias 5%**	$8.53x10^{6}$	$8.55x10^{6}$	$8.55x10^{6}$
**Codon Bias 10%**	$8.53x10^{6}$	$8.55x10^{6}$	$8.56x10^{6}$
**Codon Bias 15%**	$8.53x10^{6}$	$8.55x10^{6}$	$8.55x10^{6}$
**Codon Bias 20%**	$8.57x10^{6}$	$8.59x10^{6}$	$1.62x10^{8}$

Table SI3: Objective function values for diversification and similarity runs using the ABCD approach without exclusion of target sequences except for bacterial IRBs.

Supplementary Note 1: Codon diversified sequences for proteins in this study

cdCitrine(M20)

GAATTCAGCAAGAAAATGGTCTCCAAAGGAGAGGAACTGTTTACAGGCGTGGTCCCTATTCTGGT
GGAACTGGATGGAGATGTCAATGGACACAAATTTTCCGTGAGCGGAGAAGGGGAAGGAGATGCT
ACTTATGGAAAACTGACTCTGAAATTTATTTGTACTACAGGGAAACTGCCAGTCCCATGGCCTAC
ACTGGTGACTACTTTTGGGTATGGACTGATGTGTTTTGCTAGGTATCCAGATCACATGAAACAAC
ATGACTTTTTTAAAAGCGCTATGCCAGAAGGATATGTGCAAGAAAGAACTATTTTTTTCAAAGATG
ATGGAAATTATAAGACTAGAGCAGAGGTCAAGTTTGAGGGAGATACTCTGGTCAATAGAATTGAA
CTGAAAGGAATTGATTTTAAGGAAGATGGGAACATTCTGGGACATAAACTGGAATACAATTATAAC
TCCCACAATGTGTATATTATGGCAGACAAACAGAAAAATGGAATTAAAGTGAATTTTAAGATTAGA
CATAATATCGAAGACGGAAGCGTCCAACTGGCTGATCATTATCAACAAAATACACCAATCGGAGA
TGGACCTGTCCTGCTGCCTGACAATCACTATCTGTCCTATCAATCCGCACTGTCCAAGGATCCAA
ATGAGAAAAGAGACCACATGGTGCTGCTGGAATTTGTCACAGCCGCAGGAATCACACTGGGAAT
GGATGAACTGTATAAATAAGGCGCGCCACTAGTTACGTAAAGCTTC

= Linkers with restriction site cassettes

A = cdCitrine(M20)

cdcp173tdVenus(M15)

TCCATGGATGGAGGCGTTCAACTCGCAGACCATTATCAACAAAATACACCAATAGGTGATGGACC
TGTTCTCCTCCCAGATAATCATTATCTCTCATATCAAAGTGCTCTGAGTAAGGATCCAAATGAAAA
GAGAGACCACATGGTTCTGCTCGAATTTGTTACCGCTGCAGGTATAACACTCGGAATGGATGAAC
TGTATAAAGGTGGAAGTGGTGGTATGGTTTCTAAAGGGGAAGAACTCTTCACAGGAGTTGTTCCT
ATACTCGTGGAACTCGATGGAGATGTCAATGGACATAAATTTTCTGTTAGTGGAGAGGGAGAAGG
TGATGCTACTTATGGTAAACTCACACTCAAACTGATATGTACTACAGGAAAACTGCCTGTCCCTTG
GCCTACTCTCGTCACTACACTGGGTTATGGACTCCAATGTTTTGCTAGGTATCCTGATCACATGA
AACAACATGACTTTTTTAAGAGTGCAATGCCAGAAGGATATGTTCAAGAAAGAACAATATTTTTCA
AAGATGATGGTAATTATAAAACTAGGGCTGAGGTTAAATTTGAAGGAGATACTCTGGTTAATAGAA
TTGAACTGAAAGGAATAGATTTTAAAGAAGACGGAAATATACTCGGTCATAAACTGGAATATAACT
ATAATAGTCATAATGTGTATATTACTGCTGATAAACAAAAAAATGGAATTAAAGCTAATTTTAAAAT
AAGACACAATATAGAGCCGAATTTTGTCTTCCTGATCGGCGCCGCAGGTATACTCTTCGTATCTA
GCGAGCTCATGGTTAGTAAAGGAGAGGAACTGTTTACAGGTGTGGTTCCAATTCTGGTTGAACTG
GATGGTGATGTGAATGGTCATAAGTTTAGTGTCAGTGGGGAAGGAGAAGGAGACGCAACCTATG

GGAAACTGACTCTGAAACTCATCTGTACTACTGGAAAACTCCCTGTTCCTTGGCCAACACTGGTT
ACAACCCTGGGATACGGTCTCCAATGCTTTGCAAGATACCCTGACCACATGAAACAGCATGATTT
TTTTAAATCTGCTATGCCTGAAGGTTACGTTCAGGAAAGAACTATATTTTTTAAAGACGATGGCAA
TTATAAGACTAGAGCCGAAGTGAAATTTGAGGGTGATACACTCGTTAATAGGATAGAACTCAAAG
GTATTGATTTTAAGGAAGATGGAAACATACTGGGACATAAACTCGAATACAATTACAATTCACATA
ATGTTTATATCACTGCAGACAAACAAAAGAATGGTATAAAAGCAAACTTTAAGATTAGACATAATAT
CGAAGACGGTGGAGTTCAACTGGCTGATCATTACCAACAGAATACTCCTATCGGTGACGGACCA
GTTCTCCTGCCAGACAATCACTATCTCAGTTATCAGTCAGCACTCAGTAAAGATCCTAATGAAAAA
AGAGATCACATGGTGCTCCTCGAGTTTGTGACAGCCGCAGGGATAACTCTGGGTATGGATGAGC
TCTACAAAGAATTC

= Linkers with restriction site cassettes

= cdcp173Venus(M15)

A = cdVenus(M15)

cdhERT2(M15)

GCAGGGGATATGAGAGCCGCTAATCTGTGGCCCTCACCTCTGATGATAAAACGGAGTAAGAAAA
ACTCACTCGCACTCAGTCTCACAGCTGATCAAATGGTTAGTGCTCTCCTGGACGCAGAACCTCCT
ATTCTGTATAGTGAATACGATCCCACTAGACCTTTTTCAGAAGCATCTATGATGGGACTGCTCACA
AATCTCGCTGACAGAGAACTCGTTCATATGATTAATTGGGCAAAAAGAGTTCCCGGATTCGTTGA
TCTGACTCTGCACGATCAAGTTCATCTGCTCGAGTGTGCATGGCTGGAAATACTCATGATAGGAC
TGGTTTGGAGAAGTATGGAACATCCAGTTAAACTCCTCTTTGCACCTAATCTCCTCCTCGATAGAA
ATCAAGGAAAGTGTGTTGAAGGTATGGTTGAAATTTTTGATATGCTCCTCGCAACTAGTAGTAGAT
TTAGAATGATGAACCTCCAAGGTGAAGAATTTGTTTGTCTCAAGTCAATTATTCTGCTGAATAGTG
GTGTTTATACTTTCCTGTCTAGTACTCTGAAATCACTCGAAGAAAAAGATCATATACATAGAGTTC
TCGATAAAATAACAGATACTCTCATTCATCTCATGGCTAAGGCCGGTCTGACTCTCCAACAACAA
CATCAAAGACTCGCTCAACTGCTCCTGATACTGTCACATATAAGACATATGAGCAATAAAGGTAT
GGAACATCTCTATAGTATGAAATGTAAAAATGTCGTTCCACTCTACGATCTCCTGCTCGAGGCTG
CCGATGCACATAGACTGCACGCACCTACTTCTAGGGGTGGAGCCAGTGTTGAAGAAACTGATCA
ATCTCATCTGGCAACAGCTGGTTCTACAAGTAGTCATAGTCTCCAAAAATATTATATAACTGGAGA
AGCTGAGGGATTTCCAGCTACT
