# Supplementary material for: Abundance-biased codon diversification prevents recombination in AAV production and ensures robust in vivo expression of functional FRET sensors
Source: Commun Biol. 2025 Aug 19;8:1244. doi: 10.1038/s42003-025-08677-6 (PMC12365220; doi:10.1038/s42003-025-08677-6)
Supplement: Supplementary file 2 — Description of Additional Supplementary Files [file 42003_2025_8677_MOESM2_ESM.docx]

Description of Additional Supplementary Files

**File name:** Supplementary Data 1

**Description:** Raw data for the plots shown in Main and Supplementary Figures
